# [Retracted] CCL26 regulates the proportion of CD4+CD25+FOXP3+ Tregs and the production of inflammatory factors in peripheral blood mononuclear cells following acute ischemic stroke via the STAT5 pathway

**DOI:** 10.3892/etm.2023.11873

**Published:** 2023-03-06

**Authors:** Zhiqiang Dong, Limei Cao, Lan Guo, Yuan Hong, Jinxiu Cao, Xu Chen

Exp Ther Med 20:3343–3351, 2020; DOI: 10.3892/etm.2020.9046

Following the publication of this paper, it was drawn to the Editor’s attention by a concerned reader that certain of the GAPDH control western blotting data shown in Fig. 4C were strikingly similar to data appearing in different form in Figs. 4E and 5 of another article written by different authors at different research institutes, which had already been submitted for publication at the time of this paper’s receipt at the *Experimental and Therapeutic Medicine* Editorial Office [Zhou Y, Hua Z, Zhu Y, Wang L, Chen F, Shan T, Zhou Y and Dai T: Upregulation of ARHGAP30 attenuates pancreatic cancer progression by inactivating the β-catenin pathway. Cancer Cell Int 20: 225, 2020].

Owing to the fact that the contentious data in the above article had already been submitted for publication prior to its submission to *Experimental and Therapeutic Medicine,* the Editor has decided that this paper should be retracted from the Journal. The authors were asked for an explanation to account for these concerns, but the Editorial Office did not receive a reply. The Editor apologizes to the readership for any inconvenience caused.

